# Comprehensive Resources for Tomato Functional Genomics Based on the Miniature Model Tomato Micro-Tom 

**DOI:** 10.2174/138920208786241225

**Published:** 2008-11

**Authors:** C Matsukura, K Aoki, N Fukuda, T Mizoguchi, E Asamizu, T Saito, D Shibata, H Ezura

**Affiliations:** 1 Graduate School of Life and Environmental Sciences, University of Tsukuba, Tennoudai, Tsukuba, 305-8572, Japan; 2 Kazusa DNA Research Institute, Kazusa-Kamatari, Kisarazu, 292-0818, Japan

**Keywords:** Expressed sequence tag, full-length cDNA, genome sequence, mutant resource, Micro-Tom, JSOL, *Solanum lycopersicum*, tomato.

## Abstract

Tomato (*Solanum lycopersicum* L., Solanaceae) is an excellent model plant for genomic research of solanaceous plants, as well as for studying the development, ripening, and metabolism of fruit. In 2003, the International Solanaceae Project (SOL, www.sgn.cornell.edu ) was initiated by members from more than 30 countries, and the tomato genome-sequencing project is currently underway. Genome sequence of tomato obtained by this project will provide a firm foundation for forthcoming genomic studies such as the comparative analysis of genes conserved among the Solanaceae species and the elucidation of the functions of unknown tomato genes. To exploit the wealth of the genome sequence information, there is an urgent need for novel resources and analytical tools for tomato functional genomics. Here, we present an overview of the development of genetic and genomic resources of tomato in the last decade, with a special focus on the activities of Japan SOL and the National Bio-Resource Project in the development of functional genomic resources of a model cultivar, Micro-Tom.

## INTRODUCTION

The Solanaceae family comprises many agriculturally valuable crops, including eggplant, potato, pepper, tobacco, and tomato. Among them, tomato is one of the most important crops in the fresh vegetable market and the food-processing industry [1]. For genetic and genomic studies, tomato has many advantages over other Solanaceae, such as the moderate size of its diploid genome (950 Mb, n = 12), having numerous mapped traits, developed DNA markers, abundant collections of germplasm and mutants, and an increasing number of expressed sequence tags (ESTs) [2-6]. These advantages have made tomato an excellent model organism for investigating fruit development [7], ripening processes [8-11], sugar metabolism [12-14], carotenoid biosynthesis [15, 16] in a fleshy berry-type fruit, quantitative trait locus (QTL) analyses [17, 18], and plant–pathogen interactions [19, 20]. For these reasons, tomato was chosen for the genome sequencing as a model species of the Solanaceae family.

The genome structures of most of the solanaceous plants are relatively well conserved [21]. Thus, the tomato genome sequence will serve as a reference to study the evolution of sequence and function of orthologous genes of solanaceous plants, which then allows researchers to investigate molecular mechanisms underlying diversification and adaptation. Additionally, a large-scale analysis of tomato ESTs revealed that approximately 30% of tomato genes do not have significant similarity with *Arabidopsis* genes [4]. Functional analysis of these genes will provide novel insights into the mechanisms controlling biological functions that are unique to tomato. To achieve these goals, it is necessary to develop resources and analytical tools for tomato functional genomics.

Small organisms with short generation time have been the choice for model systems in functional genomics, as exemplified by *Drosophila* and *Arabidopsis*. From this point of view, a miniature tomato cultivar ‘Micro-Tom’ attracts an attention as a model cultivar for tomato genomics. Micro-Tom can grow at high density in an *Arabidopsis*-like manner, which allows large-scale production of mutagenized lines. Since EMS mutagenized lines and transposable element-based enhancer- and gene-trap lines of Micro-Tom were reported [22], potential of Micro-Tom as a genomic tool has been recognized widely, and various resources for tomato functional genomics has been developed in Micro-Tom-background.

In this review, we first describe the current status of tomato genomics, with the summary of the development of genetic and genomic resources. We then focus on the genomic resources developed by using Micro-Tom, and describe the activities of Japan SOL (JSOL) and the National Bio-Resource Project in organizing the Micro-Tom genomic resources. 

## CURRENT STATE OF TOMATO GENOMICS

Tomato genomic resources have been developed in the last two decades in the form of linkage maps with various markers, ESTs, full length cDNA sequences, gene expression profiles, and genome sequences with annotations. Currently, most information from these resources has been released to databases in the public domain such as the National Center for Biotechnology Information (NCBI), the DNA Data Bank of Japan (DDBJ), the Solanaceae Genome Project Network (SGN), and the J. Craig Venter Institute (JCVI, formed through the merger of several organizations including The Institute for Genomic Research, TIGR). The current status of these tomato genomics databases has been reviewed by [23].

### Genome Sequencing by SOL

SOL started genome sequencing of 12 tomato chromosomes in 2004, focusing on 220 Mbp gene-rich euchromatic regions [24, 25]. By this approach, SOL predicts that approximately 87% of a total of 35,000 genes [4] could be sequenced, although the euchromatic region only covers 23% of all chromosomes. The progress of the project and the sequence information are provided on the SGN website (http://soldb.cit.cornell.edu/about/tomato_sequencing.pl). Prior to this project, a bacterial artificial chromosome (BAC) library was constructed with the tomato cultivar Heinz 1706 [26]. This library is composed of approximately 129,000 clones containing *Hin*dIII, *Mbo*I and *Eco*RI-digested mega-size DNAs. The average insert size of the BAC clones is 117.5 Kbp and the BAC library covers the haploid genome by 15-fold. The 88,642 BACs were fingerprinted and anchored to the high-density genetic linkage map “F2-2000” to generate a physical map [24]. Seed BACs on each chromosome were anchored to mapped markers by hybridizing oligo DNA probes developed from marker sequences to BAC-arrayed filters. Thirty to sixty seed points per chromosome were selected in this way, and BAC-by-BAC sequencing is in progress. Currently the project goal is to sequence 2,500 BACs in total to cover the estimated 220 Mbp euchromatin. The sequencing proceeds on a clone by clone basis in the ten participating countries (China, France, India, Italy, Korea, Japan, Netherlands, Spain, USA, and UK). Using the fingerprint contig physical map and a BAC end sequence database, which were constructed as a part of the project, the BAC sequences are assembled together in the euchromatin tiling path. By May 3 2008, 28.1% of the sequencing was completed. The number of genome survey sequences (GSS) accumulating in the International Nucleotide Sequence Databases (INSD) is rapidly increasing with the progress of the project. By Apr. 25, 2008, it constitutes 319,461 sequences, which is the sixth largest found among plants.

### Genetic Linkage Maps and DNA Markers 

SGN provides information for the high-density genetic linkage map of the tomato genome with DNA markers, including cleaved amplified polymorphic sequences (CAPS), restriction fragment length polymorphisms (RFLPs), single nucleotide polymorphisms (SNPs), and simple sequence repeats (SSRs;  http://soldb.cit.cornell.edu/cview/) (Table **1**). Currently, five maps constructed using segregation populations and inbred lines derived from crossing between cultivars and wild or wild derivatives are available. “Tomato-EXPEN 1992” is based on *S. lycopersicum* (cv. VF36) x *S. pennellii* (LA716) F_2_ population, including 1,005 of the DNA markers (RFLP and CAPS) and also some isozyme and morphological markers [2]. “Tomato-EXHIR 1997” is derived from the interspecific backcross of *S. lycopersicum* (TA209) x *S. habrochaites* (also known as *S. hirsutum*; LA1777), including 135 RFLP markers [27]. “Tomato-EXPEN 2000” is based on 80 F_2_ individuals from the crossing of *S. lycopersicum* (LA925) x *S. pennellii* (LA716), including 2,586 CAPS, RFLP, SNPs, and SSR markers. This map is also assisted by conserved ortholog set (COS) markers generated by a comparison of the tomato EST database to the *Arabidopsis* genome [28]. “Tomato-EXPIMP 2001” is based on three populations derived from the crossing of *S. lycopersicum *(cv. E6203) x *S. pimpinellifolium* (LA1589) BC_1_, BC_2_ and backcross recombinant inbred lines (BCRILs), including 144 CAPS and RFLP markers [21, 29, 30]. “Tomato-EXPIMP 2008” is based on *S. lycopersicum* (TA492) x *S. pimpinellifolium* (LA1589), which includes 181 CAPS, RFLP, and SSR markers. The physical maps showing the anchored positions of the BACs used for genome sequencing and the introgression line (IL) maps based on EXPEN1992 and EXPEN2000 are also available on the SGN website. These ILs were developed by successive backcrosses, and each line carries a genetically defined chromosome segment derived from *S. pennellii* (LA716) in the background of *S. lycopersicum* cv. M82 (LA3475; [31]).

### Expressed Sequence Tags, Full-Length cDNA, and Gene Expression Data

ESTs provide comprehensive information reflecting gene expression patterns in certain tissues/organs at various developmental stages, as well as sequence information. Thus far (Apr 25, 2008), 257,940 tomato EST sequences have been deposited in the INSD, the largest such database among vegetable crops (tenth in plants). These ESTs were assembled into non-redundant consensus sequence sets called ‘unigenes’ or “tentative consensus” (TC). SGN [25], the DFCI Tomato Gene Index (http://compbio.dfci.harvard.edu/tgi/cgi-bin/tgi/gimain.pl?gudb=tomato; [32]), and the MiBASE (http://www.kazusa.or.jp/jsol/microtom/indexe.html;  [33]) provide ESTs and unigene sequences on the basis of their own *in silico* construction methods. To date, SGN delivers 34,829 unigenes assembled from 223,441 ESTs (Tomato 200607), the Tomato Gene Index delivers 41,425 unigenes assembled from 215,990 ESTs (Release 11.0, June 21, 2006), and the MiBASE delivers 26,363 unigenes assembled from 186,405 ESTs, including 36,427 in-house generated ESTs (ver. Mar. 2007). These databases also support corresponding gene ontology (GO) terms, predicted peptide sequences, and DNA marker information. As described above, SGN has mapped these ESTs as COS markers on the “Tomato EXPEN2000” based on comparisons to the *Arabidopsis* genome to estimate the level of genome synteny between tomato and *Arabidopsis*. By using these resources, the following DNA microarrays have been developed: the Tom1 12K cDNA array and the Tom2 11K oligo array offered by the Center for Gene Expression Profiling (CGEP), and the GeneChip® Tomato Genome Array from Affymetrix, Inc. (Santa Clara, CA, USA). These include more than 10,000 probes designed on the basis of information from UniGene Build #20 (3 October 2004) and GenBank mRNA (5 November 2004). The Tomato Expression Database collects and releases gene expression data obtained from DNA microarray experiments with Tom1, Tom2 and the Affymetrix GeneChip on the website (http://ted.bti.cornell.edu/). This database is a part of the tomato genome project, and contains basic microarray information including SGN-supported probe sequences and annotation information as well as microarray data for fruit development [11, 34-36]. Several other databases, including ArrayExpress [37] and MiBASE [33, 38, 39], also have open gene expression data obtained from microarray experiments. These databases will continue to be updated with additional expression results. Aside from ESTs and unigenes, full-length cDNA libraries derived from fruit and leaves, including pathogen-treated tissue, have been developed [40] and their sequence information is released to INSD and KaFTom (http://www.pgb.kazusa.or.jp/kaftom/). Together with the comprehensive gene expression analysis data, full-length cDNA resource accelerates the application of reverse-genetics approach to elucidate the function of tomato genes.

### Collection of Tomato Germplasm Stock 

One direct way to identify a gene and understand its function involves a forward genetic approach based on mutation analysis. Currently, 1,017 monogenic mutants at 622 loci have been collected in the Tomato Genetics Resource Center (TGRC) at the University of California, Davis (http://tgrc.uctavis.edu). These monogenic mutants contain spontaneous and induced mutations affecting many aspects of plant development. In addition to the mutants, TGRC has stored wild relatives, including representatives of all nine *Lycopersicon* species, four related *Solanum* species, and approximately 1,500 miscellaneous genetic stocks including landraces, cultivars, prebred lines, introgression lines, backcross recombinant inbred lines, stress-tolerant stock, and cytogenetic stock containing trisomics, tetraploids, and translocations. Except for rare stock with low fertility, TGRC distributes the seed stock gratis to researchers for research purposes. Additionally, an isogenic mutation library containing more than 3,400 mutations has been developed in the genetic background of the inbred variety ‘M82’ and cataloged in the SGN on a site named “Genes That Make Tomatoes” (http://Zamir.sgn.cornell.edu/mutants/; [5]). However, since a current rough estimation suggests that the number of genes in the tomato genome ranges from 30,000 to 35,000, the current mutant population is insufficient for saturated mutagenesis. Considerable efforts have been made to generate a large M_2_ population through chemical (e.g., ethyl methane sulfonate; EMS), physical (e.g., X-ray or fast-neutron irradiation), and insertional (e.g., transposable elements or T-DNA) mutagenesis [5, 22, 41-45].

## MODEL CULTIVAR MICRO-TOM FOR FUNCTIONAL GENOMICS

### cv. Micro-Tom

The miniature and dwarf *S. lycopersicum* cultivar Micro-Tom (TGRC accession # LA3911) was bred for home gardening purposes by crossing cv. Florida Basket and Ohio 4013-3 [46]. From the late 1990s, the Micro-Tom has received attention as a model cultivar for molecular research on tomato. Compared to other cultivars, it has several unique features, such as small plant size (15–20 cm high), rapid life cycle (70–90 days), and easy transformation [22, 45]. It also exhibits relatively high fertility and fruit set even under normal fluorescent lighting. Meissner *et al.* [22] showed that it can be grown at high density (up to 1,357 individuals/m^2^) and can produce three or four generations in a year. These utilities, which most ordinary tomato cultivars lack, allow us to handle this cultivar in an *Arabidopsis*-like manner, making it successful for large-scale and high-throughput work in functional genomics.

The miniature growth phenotype of Micro-Tom is attributed to at least two major recessive mutations, *dwarf *(*d*) and *miniature *(*mnt*) derived from its ancestors [22]. The *D* gene encodes cytochrome P450 protein, which is a brassinosteroid biosynthetic enzyme [47]. Reduced brassinosteroid content in Micro-Tom results in its rugose deep-green leaves and shortened internode phenotype [48]. Although the *mnt* mutation has not been well characterized, it is suggested to be associated with gibberellin (GA) signaling without affecting GA metabolism [48]. A mutation in the *SELF PRUNING *(*SP*) gene is responsible for the determinate phenotype of Micro-Tom [48]. *SP *is an ortholog of the *Arabidopsis* *TFL1 *(*TERMINAL FLOWER 1*) gene, which is involved in continuous growth of the shoot apical meristem [49]. 

### Micro-Tom Mutagenized Lines

During the last decade, a large number of mutagenized population resources have been developed in Micro-Tom: *Ac/Ds* transposon insertional tagging lines and T-DNA insertional activation tagging lines, EMS-mutagenized lines, and gamma irradiation mutagenized lines. The transposon tagging lines proved that an *Ac/Ds* system derived from maize is active in the Micro-Tom genome. A total of 2,932 lines harboring two to three *Ds* elements (approximately 7,500 insertions in total) were generated, and the *Ds* insertion was preferentially inserted into genes [50]. A novel transcription factor, *ANT1*, which was isolated from a population of 10,427 independent T-DNA tagging lines, encodes a MYB transcription factor regulating gene expression of a group of anthocyanin biosynthetic enzymes in tomato [51]. These results show the feasibility of insertional mutagenesis as a reverse genetic approach *via *a knockout strategy, as found in other tomato cultivars [47, 52, 53]. However, based on the number of genes in the tomato genome, it is estimated that 200,000–300,000 lines harboring two or three insertions in each line will be necessary for construction of a population containing sufficient mutations to knock out a target gene [50]. This scale requirement would be a disadvantage for insertional mutagenesis strategies. International collaborations will be necessary for the saturated mutagenesis of tomato by such approaches.

In tomato, EMS has frequently been used to induce point mutations. One of the big advantages of EMS mutagenesis is that it causes high-frequency and wide-spectrum mutations in the entire genome. This means that near-saturated mutagenesis can be expected with a relatively small population (several 10^3^–10^4^ lines). To date, several groups, including our own, have generated EMS mutagenized populations and recovered a broad range of mutant alleles [22, 54 and C. Rothan, personal communication]. Recently, a highly sensitive point mutation detection technique, named Targeting Induced Local Lesions IN Genomes (TILLING), was developed and is available for use in plants to identify a point mutation [55, 56]. By constructing a system combining EMS mutagenized resources and TILLING, we can expect to obtain a desirable mutant through large-scale and high-throughput screening. A gamma-ray (γ -ray) irradiated M_2_ population was also generated in Micro-Tom, and several dozen of severe phenotypic mutants have been obtained to date through consequent screening [57]. Such a mutation resource caused by large-size deletions would be applicable for reverse genetic approaches supported by PCR-based screening methods as well as for forward genetics approaches, as established in *Arabidopsis* and rice [44]. Considering the requirements for regulatory approval of genetically modified organisms (GMOs), non-transgenic mutant resources will be preferred as parental germplasm for a breeding program. Overall, all mutagenesis strategies reported in other cultivars and species are also applicable to Micro-Tom. The unique features of this cultivar are very profitable for taking advantage of scale to reach saturated mutagenesis in tomato.

### Development of a High-Efficiency Transformation System with Micro-Tom

Efficient genetic transformation is an essential technology for functional genomics. This experimental tool is invaluable to elucidate and verify the function of a target gene through transgenic plants, T-DNA insertional tagging lines, and complementation analyses. Although *Agrobacterium*-mediated transformation is applicable to *S. lycopersicum* [58], its efficiency had been relatively lower than that of other model species such as *Arabidopsis* and rice. High transformation frequencies of up to 60–80% have been reported in tomato [22, 51], but there are no detailed descriptions of the transformation methods. According to other early studies, the efficiencies range from 6 to a maximum of 37% [59-65]. Furthermore, large plant size and long life cycle (120–150 days) of ordinary tomato cultivars has discouraged researchers from scale expansion of transformation experiments, which require a large-scale facility which meets the regulations for GMOs. Recently, we and another group independently developed a highly efficient transformation protocol for Micro-Tom to break this stalemate [66, 67]. The protocols use cotyledons as a starting explant, and the transformation efficiency based on independent transgenic events per inoculated explant stably exceeded 40% in the study by [67] and ranged from 24 to 80% (on average 56%) in the study by [66]. Our group modified and refined a protocol that had been reported for other tomato cultivars [13]. In this protocol, the efficient elimination of chimeric status by repeated shoot elongation on the selection medium is critical for efficient generation of stable transgenic plants. [66] also made modifications to existing protocols; they indicated that optimal initial infection of *Agrobacterium* is critical for high frequency and stable transformation, and suggested to use the figwort mosaic virus (FMV) promoter [68] to drive target gene. It is noteworthy that both methods enabled us to obtain a transgenic plant in about 3 months after *in vitro* seed germination for explant preparation. These protocols for Micro-Tom will provide a powerful tool for functional genomics of tomato.

### Application for Pathology

Tomato is a well-established model plant to investigate interactions between plants and pathogens. Tomato is susceptible to a wide range of plant diseases induced by over 60 fungi, bacteria, viruses, viroids, and nematodes [69]. On the other hand, over 20 disease-resistance loci have been identified, and these traits have been introduced into *S. lycopersicum* cultivars from various wild species [70]. Micro-Tom is susceptible to at least four fungal and two bacterial species and two viruses [71]. It also exhibits resistance to *Fusarium* wilt race 1 and grey leafspot, which were derived from an original variety [46]. Additionally, [48] reported that Micro-Tom is resistant to tomato leaf mold caused by *Cladosporium fulvum*. However, the resistance does not correspond to the previously reported *Cf-2*, *Cf-4*, *Cf-5*, and *Cf-9* genes, which control resistance to various races of *C. fulvum* in a dose-dependent manner [52, 72-74]. Because many uncharacterized *Cf*-like genes in the tomato genome have been reported [75], Micro-Tom represents a new target cultivar to study resistance to tomato leaf mold. The unique features of Micro-Tom, increasing genomic resources, and sequence information will facilitate its use as a model plant in the field of plant pathology.

### Micro-Tom Resource Development by Japanese Solanaceae Research Community

**Fig. (1):**
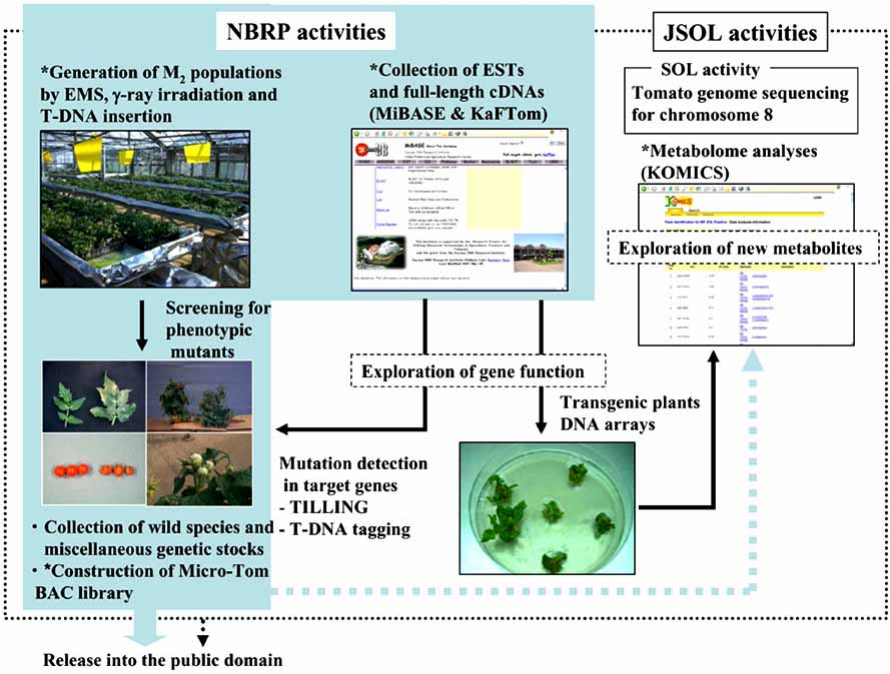
Current state of tomato functional genomics in Japan through the JSOL and NBRP activities. The activities based on Micro-Tom are marked with asterisks.

Japan has contributed to the SOL genome sequencing project through JSOL activities. JSOL was voluntarily formed in 2004 by Japanese tomato-related researchers from universities, public research organizations and private companies. Alongside the efforts in genome sequencing, we have developed the tomato genomic resources based on Micro-Tom: i) collection of ESTs and full-length cDNAs; ii) construction of BAC libraries (in progress); iii) generation of mutagenized M_2_ populations by EMS and γ-ray irradiation and consequent mutant screening [54, 57]; iv) establishment of advanced tools for functional genomics research, including the high-throughput genetic transformation protocol [67], DNA arrays [38], the TILLING screening system and T-DNA-insertional tagging lines; and v) database construction of these resources in the public domain (Fig. **1**). To date (April 25 2008), we have obtained 36,427 ESTs and 2,268 putative full-length cDNA sequences derived from fruit and leaves, including from pathogen-treated tissues [6, 40]. Information on these resources has been released on the Kazusa DNA Research Institute websites, MiBASE and KaFTom, with DNA array profiling data [33, 39]. We also have a total stock of 10,267 M_2_ families (EMS mutagenesis 3,845; gamma irradiation mutagenesis 6,422, as of 3 May 2008), and 489 phenotypically categorized definitive mutants screened from the populations [54, 57]. We have already shared these genomic resources among JSOL members. The Kazusa DNA Research Institute has released a large part of the resources and information into the public domain since 2005. Additionally, from 2008, our group (University of Tsukuba) will begin sharing the EMS-mutagenized M_2_ resources with the French National Institute for Agricultural Research (INRA), which has already generated approximately 8,000 M_2_ populations in Micro-Tom and concomitantly established a TILLING screening system (C. Rothan, personal communication). This international research collaboration will greatly facilitate comprehensive genomic analyses for exploring gene function by taking advantage of the scale of the program.

In 2007, tomato was designated as a national biological resource by the National Bio-Resource Project (NBRP) funded by the Ministry of Education, Culture, Sports, Science and Technology (MEXT), Japan. The University of Tsukuba and the Kazusa DNA Research Institute are the core organizations in this tomato project, and the above-mentioned genomic resources are core project resources. During the next 10 years, we will continue to collect and distribute tomato genomic resources based on Micro-Tom. We will also develop and improve new bioresources and concomitant analytical tools, including large-scale T-DNA tagging lines and mapping populations, a reverse-genetic screening system, and metabolome databases (http://webs2.kazusa.or.jp/komics/index.php) [76], through the activities of JSOL and NBRP (Fig. **1**).

## Figures and Tables

**Table 1.** Tomato Genetic Linkage Maps Provided by the Solanaceae Genome Project Network (SGN)

Map Name	Cross Combination	Population	References
Tomato-EXPEN 1992	*S. lycopersicum* (cv. VF36) x *S. pennellii* (LA716)	F_2_	[2]
Tomato-EXHIR 1997	*S. lycopersicum* (TA209) x *S. habrochaites* (LA1777)	BC_1_^x^	[27]
Tomato-EXPEN 2000	*S. lycopersicum* (LA925) x *S. pennellii* (LA716)	F_2_	[2, 28]
Tomato-EXPIMP 2001	*S. lycopersicum* (cv. E6203) x *S. pimpinellifolium* (LA1589)	BC_1_, BC_2_ and BCRILs^y^	[21, 29, 30]
Tomato-EXPIMP 2008	*S. lycopersicum* (TA492) x *S. pimpinellifolium* (LA1589)		
Introgression line (IL) map based on the EXPEN 1992	*S. lycopersicum* (cv. M82) x *S. pennellii* (LA716)	NILs^z^	[31]
Introgression line (IL) map based on the EXPEN 2000	*S. lycopersicum* (cv. M82) x *S. pennellii* (LA716)	NILs	[31]

x, y, z means backcross, backcross recombinant inbred lines and nearly isogenic lines, respectively.

## ABBREVIATIONS

BAC: = Bacterial artificial chromosome
CAPS: = Cleaved amplified polymorphic sequences
EMS: = Ethyl methane sulfonate
EST: = Expressed sequence tag
JSOL: = Japan SOL
QTL: = Quantitative trait locus
RFLP: = Restriction fragment length polymorphism
SGN: = Solanaceae genome project network
SNPs: = Single nucleotide polymorphisms
SOL: = International Solanaceae Project
SSR: = Simple sequence repeat
TGRC: = Tomato genetics resource center
TIGR: = Institute for genomic research
TILLING: = Targeting induced local lesions in genomes

## References

[1] Rick CM, Yoder JI (1988). Classical and molecular genetics of tomato: highlights and perspectives. Annu. Rev. Genet.

[2] Tanksley SD, Ganal MW, Prince JP, de Vicente MC, Bonierbale MW, Broun P, Fulton TM, Giovannoni JJ, Grandillo S, Martin GB, Messeguer R, Miller JC, Miller L, Paterson AH, Pineda O, Roder MS, Wing RA, Wu W, Young ND (1992). High density molecular linkage maps of the tomato and potato genomes. Genetics.

[3] Tanksley SD, O'Brien J (1993). Linkage map of tomato (*Lycopersicon esculentum*) (2N=24). Genetic Maps: Locus Maps of Complex Genomes.

[4] Van der Hoeven R, Ronning C, Giovannoni J, Martin G, Tanksley S (2002). Deductions about the number, organization, and evolution of genes in the tomato genome based on analysis of a large expressed sequence tag collection and selective genomic sequencing. Plant Cell.

[5] Menda N, Semel Y, Peled D, Eshed Y, Zamir D (2004). In silico screening of a saturated mutation library of tomato. Plant J.

[6] Yamamoto N, Tsugane T, Watanabe M, Yano K, Maeda F, Kuwata C, Torki M, Ban Y, Nishimura S, Shibata D (2005). Expressed sequence tags from the laboratory-grown miniature tomato (*Lycopersicon esculentum*) cultivar Micro-Tom and mining for single nucleotide polymorphisms and insertions/deletions in tomato cultivars. Gene.

[7] Gillaspy G, Ben-David H, Gruissem W (1993). Fruits: a developmental perspective. Plant Cell.

[8] Wilkinson JQ, Lanahan MB, Yen HC, Giovannoni JJ, Klee HJ (1995). An ethylene-inducible component of signal transduction encoded by never-ripe. Science.

[9] Giovannoni J (2001). Molecular biology of fruit maturation and ripening. Annu. Rev. Plant Physiol. Plant Mol. Biol.

[10] Giovannoni J (2007). Fruit ripening mutants yield insights into ripening control. Curr. Opin. Plant Biol.

[11] Alba R, Payton P, Fei Z, McQuinn R, Debbie P, Martin GB, Tanksley SD, Giovannoni JJ (2005). Transcriptome and selected metabolite analyses reveal multiple points of ethylene control during tomato fruit development. Plant Cell.

[12] Robinson NL, Hewitt JD, Bennett AB (1988). Sink metabolism in tomato fruit. I. Developmental changes in carbohydrate metabolizing enzymes. Plant Physiol.

[13] Ohyama A, Ito H, Sato T, Nishimura S, Imai T, Hirai M (1995). Suppression of acid invertase activity by antisense RNA modifies the sugar composition of tomato fruit. Plant Cell Physiol.

[14] Carrari F, Baxter C, Usadel B, Urbanczyk-Wochniak E, Zanor MI, Nunes-Nesi A, Nikiforova V, Centero D, Ratzka A, Pauly M, Sweetlove LJ, Fernie AR (2006). Integrated analysis of metabolite and transcript levels reveals the metabolic shifts that underlie tomato fruit development and highlight regulatory aspects of metabolic network behavior. Plant Physiology.

[15] Bramley PM (2002). Regulation of carotenoid formation during tomato fruit ripening and development. J. Exp. Bot.

[16] Isaacson T, Ronen G, Zamir D, Hirschberg J (2002). Cloning of tangerine from tomato reveals a carotenoid isomerase essential for production of β-carotene and xanthophylls in plants. Plant Cell.

[17] deVicente MC, Tanksley SD (1993). QTL analysis of transgressive segregation in an interspecific tomato cross. Genetics.

[18] Frary A, Nesbitt TC, Grandillo S, Knaap E, Cong B, Liu J, Meller J, Elber R, Alpert KB, Tanksley SD (2000). fw2.2: a quantitative trait locus key to the evolution of tomato fruit size. Science.

[19] Salmeron JM, Oldroyd G, Rommens C, Scofield SR, Kim H-S, Lavelle DT, Dahlbeck D, Staskawicz BJ (1996). Tomato *Prf* is a member of the leucine-rich repeat class of plant disease resistance genes and lies embedded within the *Pto* kinase gene cluster. Cell.

[20] Pedley KF, Martin GB (2003). Molecular basis of *pto*-mediated resistance to bacterial speck disease in tomato. Annu. Rev. Phytopathol.

[21] Doganlar S, Frary A, Ku HM, Tanksley SD (2002). Mapping quantitative trait loci in inbred backcross lines of *Lycopersicon pimpinellifolium* (LA1589). Genome.

[22] Meissner R, Jacobson Y, Melamed S, Levyatuv S, Shalev G, Ashri A, Elkind Y, Levy A (1997). A new model system for tomato genetics. Plant J.

[23] Yano K, Aoki K, Shibata D (2007). Genomic databases for tomato. Plant Biotechnol.

[24] Mueller LA, Solow TH, Taylor N, Skwarecki B, Buels R, Binns J, Lin C, Wright MH, Ahrens R, Wang Y, Herbst EV, Keyder ER, Menda N, Zamir D, Tanksley SD (2005a). The SOL Genomics Network: a comparative resource for Solanaceae biology and beyond. Plant Physiol.

[25] Mueller LA, Tanksley SD, Giobannoni JJ, Van Eck J, Stack S, Choi D, Kim BD, Chen M, Cheng Z, Li C, Ling H, Xue Y, Scymour G, Bishop G, Bryan G, Sharma R, Khurana J, Tyagi A, Chattopadhyay D, Singh NK, Stiekeme W, Lindhour P, Jesse T, Lankhorst RK, Bouzayen M, Shibata D, Tabata S, Granell A, Botella MA, Giuliano G, Frusciante L, Causse M, Zamir D (2005b). The Tomato Sequencing Project, the first cornerstone of the International Solanaceae Project (SOL) Comp. Comp.-Funct.-Genom.

[26] Budiman MA, Mao L, Wood TC, Wing RA (2000). A deep-coverage tomato BAC library and prospects toward development of an STC framework for genome sequencing. Genome Res.

[27] Bernacchi D, Tanksley SD (1997). An interspecific backcross of *Lycopersicon esculentum* x *L. hirsutum*: Linkage analysis and a QTL study of sexual compatibility factors and floral traits. Genetics.

[28] Fulton T, Van der Hoeven R, Eannetta N, Tanksley S (2002). Identification, analysis and utilization of conserved ortholog set (COS) markers for comparative genomics in higher plants. Plant Cell.

[29] Grandillo S, Tanksley SD (1996). QTL analysis of horticultural traits differentiating the cultivated tomato from the closely related species *Lycopersicon pimpinellifolium*. Theor. Appl. Genet.

[30] Tanksley SD, Grandillo S, Fulton TM, Zamir D, Eshed Y, Petiard V, Lopez J, Beck-Bunn T (1996). Advanced backcross QTL analysis in a cross between an elite processing line of tomato and its wild relative *L. pimpinellifolium*. Theor. Appl. Genet.

[31] Eshed Y, Zamir D (1994). A genomic library of *Lycopersicon pennellii* in *L. esculentum*: A tool for fine mapping of genes. Euphytica.

[32] Lee Y, Tsai J, Sunkara S, Karamycheva S, Pertea G, Sultana R, Antoneacu V, Chan A, Cheung F, Quackenbush J (2005). The TIGR gene indices: clustering and assembling EST and known genes and integration with eukaryotic genomics. Nucleic Acids Res.

[33] Yano K, Watanabe M, Yamamoto N, Tsugane T, Aoki K, Sakurai N, Shibata D (2006a). MiBASE: A database of a miniature tomato cultivar Micro-Tom. Plant Biotechnol.

[34] Alba R, Fei Z, Payton P, Liu Y, Moore SL, Debbie P, Cohn J, D'Ascenzo M, Gordon JS, Rose JK, Martin G, Tanksley SD, Bouzayen M, Jahn MM, Giovannoni J (2004). ESTs, cDNA microarrays and gene expression profiling: tools for dissecting plant physiology and development. Plant J.

[35] Fei Z, Tang X, Alba R, Giovannoni J (2006). Tomato Expression Database (TED): a suite of data presentation and analysis tools. Nucleic Acids Res.

[36] Moore S, Payton P, Wright M, Tanksley S, Giovannoni J (2005). Utilization of tomato microarrays for comparative gene expression analysis in the Solanaceae. J. Exp. Bot.

[37] Parkinson H, Sarkans U, Shojatalab M, Abeygunawardena N, Contrino S, Coulson R, Farne A, Garcia Lara G, Holloway E, Kapushesky M, Lilja P, Mukherjee G, Oezcimen A, Rayner T, Rocca-Serra P, Sharma A, Sansone S, Brazma A (2005). ArrayExpress-a public repository for microarray gene expression data at the EBI. Nucleic Acids Res.

[38] Yano K, Tsugane T, Watanabe M, Maeda F, Aoki K, Shibata D (2006b). Non-biased distribution of tomato genes with no counterparts in *Arabidopsis thaliana* in expression patterns during fruit maturation. Plant Biotechnol.

[39] Aoki K, Yano K, Sakurai N, Tsugane T, Watanabe M, Yin Y-G, Matsukura C, Shibata D (2007). Expression-based approach toward functional characterization of tomato genes that have no or weak similarity to *Arabidopsis* genes. Acta Hortic.

[40] Tsugane T, Watanabe M, Yano K, Sakurai N, Suzuki H, Shibata D (2005). Expressed sequence tags of full-length cDNA clones from the miniature tomato (*Lycopersicon esculentum*) cultivar Micro-Tom. Plant Biotechnol.

[41] Hildering GJ, Verkerk K (1965). Chimeric structure of the tomato plant after seed treatment with Ems and X-rays. The Use of Induced Mutations in Plant Breeding.

[42] Knapp S, Larondelle Y, Robberg M, Furtek D, Theres K (1994). Transgenic tomato lines containing *Ds* elements at defined genomic positions as tools for targeted transposon tagging. Mol. Gen. Genet.

[43] Thomas CM, Jones D, English JJ, Carroll BJ, Bennetzen JL, Harrison K, Burbidge A, Bishop GJ, Jones JDG (1994). Analysis of the chromosomal distribution of transposon-carrying T-DNAs in tomato using the inverse polymerase chain reaction. Mol. Gen. Genet.

[44] Li X, Song Y, Century K, Straight S, Ronald P, Dong X, Lassner M, Zhang Y (2001). A fast neutron deletion mutagenesis-based reverse genetics system for plants. Plant J.

[45] Emmanuel E, Levy AA (2002). Tomato mutants as tools for functional genomics. Curr. Biol.

[46] Scott JW, Harbaugh BK (1989). MICRO-TOM—a miniature dwarf tomato. Agricultural Experiment Station, Institute of Food and Agricultural Sciences, University of Florida, Circular.

[47] Bishop GJ, Harrison K, Jones JDG (1996). The tomato *Dwarf* gene isolated by heterologous transposon tagging encodes the first member of a new cytochrome *P*_450_ family. Plant Cell.

[48] Marti E, Gisbert C, Bishop GJ, Dixon MS, Garcia-Martinez JL (2006). Genetic and physiological characterization of tomato cv. Micro-Tom. J. Exp. Bot.

[49] Pnueli L, Carmel-Goren L, Hareven D, Gutfinger T, Alvarez J, Ganal M, Zamir D, Lifschitz E (1998). The *SELF-PRUNING* gene of tomato regulates vegetative to reproductive switching of sympodial meristems and is the orthologue of *CEN* and *TFL1*. Development.

[50] Meissner R, Chagua V, Zhu Q, Emmanuel E, Elkind Y, Levy AA (2000). A high-throughput system for transposon tagging and promoter trapping in tomato. Plant J.

[51] Mathews H, Clendennen SK, Caldwell CG, Liu XL, Connors K, Matheis N, Schuster DK, Menasco DJ, Wagoner W, Lightner J, Wagner DR (2003). Activation tagging in tomato identifies a transcriptional regulator of anthocyanin biosynthesis, modification and transport. Plant Cell.

[52] Jones DA, Thomas CM, Hammond-Kosack KE, Balint-Kurti PJ, Jones JDG (1994). Isolation of the tomato *Cf-9* gene for resistance to *Cladosporium fulvum* by transposon tagging. Science.

[53] Takken F, Schipper D, Nijkamp H, Hille J (1998). Identification and *Ds*-tagged isolation of a new gene at the *Cf-4* locus of tomato involved in disease resistance to *Cladosporium fulvum* race 5. Plant J.

[54] Watanabe S, Mizoguchi T, Aoki K, Kubo Y, Mori H, Imanishi S, Yamazaki Y, Shibata D, Ezura H (2007). Ethylmethanesulfonate (EMS) mutagenesis of *Solanum lycopersicum* cv. Micro-Tom for large-scale mutant screens. Plant Biotechnol.

[55] McCallum CM, Comai L, Greene EA, Henikoff S (2000). Targeting induced local lesions in genomes (TILLING) for plant functional genomics. Plant Physiol.

[56] Till JB, Reynolds HS, Weil C, Springer N, Burtner C, Young K, Bowers E, Codomo AC, Enns CL, Odden AR, Greene AE, Comai L, Henikoff S (2004). Discovery of induced point mutations in maize genes by TILLING. BMC Plant Biol.

[57] Matsukura C, Yamaguchi I, Inamura M, Ban Y, Kobayashi Y, Yin Y-G, Saito T, Kuwata C, Imanishi S, Nishimura S (2007). Generation of gamma irradiation-induced mutant lines of the miniature tomato (*Solanum lycopersicum* L. cultivar Micro-Tom. Plant Biotech.

[58] McCormick S, Niedermeyer J, Fry J, Barnason A, Horsch R, Fraley R (1986). Leaf disc transformation of cultivated tomato (*L. esculentum*) using *Agrobacterium tumefaciens*. Plant Cell Rep.

[59] Hamza S, Chupeau Y (1993). Re-evaluation of conditions for plant regeneration and *Agrobacterium*-mediated transformation from tomato (*Lycopersicon esculentum*). J. Exp. Bot.

[60] Van Roekel JSC, Damm B, Melchers LS, Hoekema A (1993). Factors influencing transformation frequency of tomato (*Lycopersicon esculentum*) expressed sequence tags from the laboratory-grown miniature tomato. Plant Cell Rep.

[61] Frary A, Earle RD (1996). An examination of factors affecting the efficiency of *Agrobacterium*-mediated transformation of tomato. Plant Cell Rep.

[62] Ling HQ, Kriseleit D, Gaal MG (1998). Effect of ticarcillin/potassium clavulanate on callus growth and shoot regeneration in *Agrobacterium*-mediated transformation of tomato (*Lycopersicon esculentum* Mill). Plant Cell Rep.

[63] Vidya CSS, Manoharan M, Kumar CTR, Savithri HS, Sita GL (2000). *Agrobacterium*-mediated transformation of tomato (*Lycopersicon esculentum* var. Pusa Ruby) with coat-protein gene of *Physalis* mottle tymovirus. J. Plant Physiol.

[64] Hu W, Phillips GC (2001). A combination of overgrowth-control and antibiotics improves *Agrobacterium tumefaciens*-mediated transformation efficiency for cultivated tomato (*L. esculentum). In Vitro*. Cell. Biol.

[65] Park SH, Morris JL, Park JE, Hirschi KD, Smith RH (2003). Efficient and genotype-independent *Agrobacterium*-mediated tomato transformation. J. Plant Physiol.

[66] Dan YH, Yan H, Munyikwa T, Dong J, Zhang B, Armstrong CL (2006). MicroTom - a high-throughput model transformation system for functional genomics. Plant Cell Rep.

[67] Sun HJ, Uchii S, Watanabe S, Ezura H (2006). A highly efficient transformation protocol for Micro-Tom, a model cultivar for tomato functional genomics. Plant Cell Physiol.

[68] Rogers SG (1990). Promoter for transgenic plants. US Patent No. 05378619.

[69] (2000). The Phytopathological Society of Japan. Common names of plant diseases in Japan. Japan Plant Protection Association, Tokyo, Japan.

[70] Arie T, Takahashi H, Kodama M, Teraoka T (2007). Tomato as a model plant for plant-pathogen interactions. Plant Biotechnol.

[71] Takahashi H, Shimizu A, Arie T, Rosmalawati S, Fukushima S, Kikuchi M, Hikichi Y, Kanda A, Takahashi A, Kiba A, Ohnishi K, Ichinose Y, Taguchi F, Yasuda C, Kodama M, Egusa M, Matsuta C, Sawada H, Shibata D, Hori K, Watanabe Y (2005). Catalogue of Micro-Tom tomato responses to common fungal, bacterial, and viral pathogens. J. Gen. Plant Pathol.

[72] Dixon MS, Jones D, Keddie JS, Thomas CM, Harrison K, Jones JDG (1996). The tomato *Cf-2* disease resistance gene comprises two functional genes encoding leucine-rich repeat proteins. Cell.

[73] Dixon MS, Hatzixanthis K, Thomas CM, Harrison K, Jones JDG (1998). The tomato *Cf-5* disease resistance gene and six homologues show pronounced allelic variation in leucine-rich repeat copy number. Plant Cell.

[74] Thomas CM, Jones D, Parniske M, Harrison K, Balint-Kurti PJ, Hatzixanthis K, Jones JDG (1997). Characterization of tomato *Cf-4* gene for resistance to *Cladosporium fulvum* identifies sequences that determine recognitional specificity in Cf-4 and Cf-9. Plant Cell.

[75] Kruijt M, De Kock MJD, De Wit PJGM (2005). Receptor-like proteins involved in plant disease resistance. Mol. Plant Pathol.

[76] Iijima Y, Nakamura Y, Ogata Y, Tanaka K, Sakurai N, Suda K, Suzuki T, Suzuki H, Okazaki K, Kitayama M, Kanaya S, Aoki K, Shibata D (2008). Metabolite annotations based on the integration of mass spectral information. Plant J.

